# Liver x receptors stimulate lipogenesis in bovine mammary epithelial cell culture but do not appear to be involved in diet‐induced milk fat depression in cows

**DOI:** 10.1002/phy2.266

**Published:** 2014-03-26

**Authors:** Kevin J. Harvatine, Yves R. Boisclair, Dale E. Bauman

**Affiliations:** 1 Department of Animal Science, Penn State University, University Park, Pennsylvania; 2 Department of Animal Science, Cornell University, Ithaca, New York

**Keywords:** Carbohydrate‐responsive element‐binding protein, conjugated linoleic acid, lipogenesis, liver x receptor, milk fat depression, retinoid x receptors

## Abstract

Milk fat synthesis of ruminants can be inhibited by intermediates of ruminal fatty acid biohydrogenation including *trans‐*10, *cis*‐12 conjugated linoleic acid (CLA). These biohydrogenation intermediates signal a coordinated downregulation of genes involved in mammary FA synthesis, transport, and esterification. We have previously reported decreased mammary expression of sterol response element‐binding protein 1 (SREBP1), SREBP1‐activating proteins, and thyroid hormone‐responsive spot 14 (S14) in the cow during diet‐induced milk fat depression (MFD), and treatment with *trans‐*10, *cis*‐12 CLA. Liver x receptors (LXR) and retinoid x receptors (RXR) regulate lipogenesis and are known to bind polyunsaturated FA and LXR agonist increases lipid synthesis in mammary epithelial cell culture. The current studies investigated if biohydrogenation products of rumen origin inhibit mammary lipogenesis through LXR and/or RXR. Expression of LXRs was not different in lactating compared to nonlactating bovine mammary tissue, and expression of LXRs, RXR*α*, and selected LXR and RXR target genes was not changed in mammary tissue during diet‐induced or CLA‐induced MFD in the cow. In bovine mammary epithelial cell culture, LXR agonist stimulated lipogenesis and expression of LXRß, ATP‐binding cassette 1 (ABCA1), SREBP1c, and S14, but LXR activation did not overcome CLA inhibition of lipogenesis and downregulation of LXRß, SREBP1c, and S14 expression. Lastly, expression of the LXR‐regulated carbohydrate‐responsive element‐binding protein (ChREBP) was higher in lactating than nonlactating tissue and was decreased during CLA‐induced MFD. We conclude that changes in mammary LXR expression in dairy cows are not involved in MFD and that *trans*‐10, *cis*‐12 CLA inhibition of lipogenesis and diet‐induced MFD appears independent of direct LXR signaling.

## Abstract (precis)

Liver x receptors (LXR) and retinoid x receptors (RXR) regulate lipogenesis and are known to bind polyunsaturated FA, and LXR agonist increases lipid synthesis in mammary epithelial cell culture. However, expression of LXRs was not different in lactating compared to nonlactating bovine mammary tissue and do not appear to be involved in trans‐10, cis‐12 CLA inhibition of lipogenesis and diet‐induced milk fat depression in the cow.

## Introduction

Mammalian milk fat concentration and composition are variable and responsive to nutritional factors. Milk fat depression (MFD) is a naturally occurring condition in ruminants fed highly fermentable diets or diets that contain plant or fish oils, and is characterized by a marked decrease in milk fat yield (Harvatine et al. 2009; Bauman et al. 2011). During diet‐induced MFD in the cow, milk fat synthesis is specifically reduced while yields of milk and other milk components are unchanged. The causative factors of MFD are specific fatty acid (FA) intermediates produced during altered ruminal biohydrogenation and *trans*‐10, *cis*‐12 conjugated linoleic acid (CLA) was the first of these unique biohydrogenation intermediates to be identified (Baumgard et al. 2000). During MFD, mammary lipogenic capacity is decreased and transcription of key genes involved in milk fat synthesis are coordinately downregulated (Harvatine et al. 2009; Bauman et al. 2011). We have previously reported decreased expression of sterol response element‐binding protein 1 (SREBP1) and thyroid hormone‐responsive spot 14 (S14) during diet‐induced and CLA‐induced MFD in the cow (Harvatine and Bauman 2006), although the mechanism by which SREBP1 and S14 are decreased has not been described. Importantly, *trans‐10, cis‐12* CLA inhibition of mammary lipid synthesis is highly conserved across species (Bauman et al. 2008).

**Figure 1.**
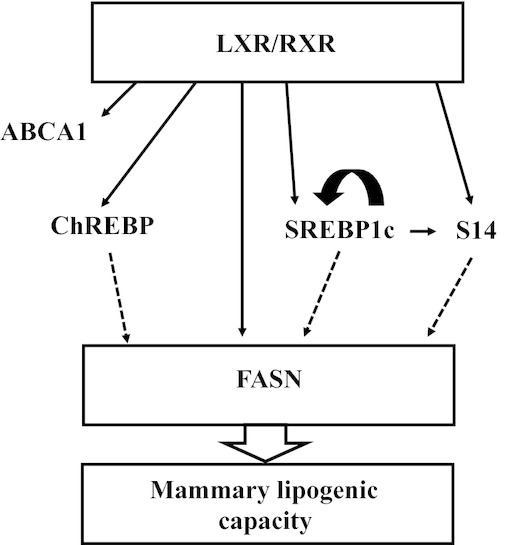
Working model of the relationship between liver x receptor (LXR) and regulation of mammary lipid synthesis tested in the current experiments. Transcriptional downregulation of lipogenic enzymes, such as fatty acid synthase (FASN), results in decreased mammary lipogenic capacity and reduced milk fat synthesis during milk fat depression in the cow. In other model systems, ligand‐activated LXR/RXR heterodimers increase expression of lipogenic enzymes such as fatty acid synthase (FASN) directly (solid arrows) and indirectly (dashed arrows) through increased expression of thyroid hormone‐responsive spot 14 (S14), sterol response element‐binding protein 1 (SREBP1), and carbohydrate response element‐binding protein (ChREBP). Additionally, secondary signals include autoregulation of SREBP1 (bold arrow) and SREBP1 regulation of S14 expression. Lastly, ABCA1 is more specific marker of LXR activity as it is predominantly regulated by LXR and is not regulated by other major lipogenesis regulators (SREBP1, S14, and ChREBP).

Some members of the nuclear hormone receptor family regulate lipogenesis and are responsive to FA in model systems (Duplus and Forest 2002; Sampath and Ntambi 2005), providing logical candidates for the mechanism of CLA‐induced MFD in the cow (Fig. 1). Specifically, liver x receptor alpha and beta (LXR*α* and LXRß) are dominant regulators of cholesterol metabolism [e.g., ATP‐binding cassette 1 (ABCA1)], but also regulate lipid synthesis directly through transcriptional activation of fatty acid synthase (FASN) and indirectly through transcriptional activation of sterol response element‐binding protein 1c (SREBP1c), thyroid hormone‐responsive spot 14 (S14), and the carbohydrate‐response element‐binding protein (ChREBP) (Yoshikawa et al. 2001; Joseph et al. 2002; Pawar et al. 2003; Cha and Repa 2007). LXR agonist stimulation of lipid synthesis in the liver is dependent on SREBP1c and ChREBP as mice lacking these genes have a muted lipogenic response to an LXR agonist (Liang et al. 2002; Cha and Repa 2007). Oxysterols are the classical natural ligand for LXRs and activation by glucose has also been described (Mitro et al. 2007). In addition, an LXR‐dependent mechanism of polyunsaturated FA (PUFA) on SREBP1c was first identified by Ou et al. (2001) and PUFA inhibition of lipogenesis has been reported to be dependent on LXR*α* in some cell lines (e.g., HEK; Pawar et al. 2002; Yoshikawa et al. 2002). Additionally, *trans*‐9, *trans*‐11 CLA was identified as a functional LXR agonist in the MCF‐7 breast cancer cells (El Roz et al. 2013). Retinoid X receptors (RXRs) are a second nuclear receptor family that functions predominantly as a heterodimeric partner with other nuclear receptors including LXRs. Retinoid X receptors are activated by 9‐*cis* retinoic acid (9cRA), although RXR*α* is also known to bind PUFA (Lengqvist et al. 2004). Importantly, RXR ligand activation stimulated lipogenesis through a SREBP1c‐dependent mechanism in HEPG2 cells (Roder et al. 2007).

The role of LXR in mammary cholesterol metabolism and lipid synthesis has been previously investigated. Modestly increased expression of LXR*α* was reported in the cow during early lactation along with expression of numerous LXR‐responsive cholesterol transporters (Mani et al. 2009, 2010). McFadden and Corl (2010) reported that LXR agonist stimulated lipid synthesis and increased expression of SREBP1 in a bovine mammary epithelial cell line (BME‐UV) and Lengi and Corl (2010) demonstrated that the response was through an LXRE in the bovine SREBP1c promoter. Lastly, Oppi‐Williams et al. (2012) reported that LXR agonist increased expression of acetyl‐CoA carboxylase and FASN independent of SREBP1 in MAC‐T cells, possibly indicating direct regulation by LXR.

Based on the ability of LXR and RXR to directly and indirectly regulate lipid synthesis (Fig. 1) and their interaction with bioactive FA, our objective was to determine the role of LXRs and RXRs in the mechanism of MFD and response to CLA in the cow. First, tissue profiling was used to investigate the tissue‐specific expression of the LXRs and ChREBP in the lactating cow. Second, expression of LXRs, RXR*α*, ChREBP, and selected target genes was observed in bovine mammary tissue during diet‐induced MFD or MFD induced by treatment with *trans*‐10, *cis*‐12 CLA. Lastly, bovine mammary epithelial cell culture was used to investigate the effect of LXR and RXR agonists on lipogenesis and gene expression and mutation of the LXR response element (LXRE) on SREBP1 expression.

## Materials and Methods

### Animal experiments

Experimental procedures involving animals were approved by the Cornell University Institutional Animal Care and Use Committee. First, tissues for analysis of spatial expression were collected from lactating cows euthanized by exsanguinated after stunning by captive bolt (subcutaneous adipose tissue, liver, skeletal muscle, heart, uterine, lung, and brain) or tissue biopsies (liver, subcutaneous adipose tissue, and mammary). Additionally, the effect of mammary physiological state was examined using mammary tissue collected from seven cows prior to lactation (approximately 30 days prepartum) and during established lactation (240 ± 85 days postpartum; mean ± SD) by needle biopsy according to Harvatine and Bauman (2006). Second, the effect of diet‐induced and CLA‐induced MFD on gene expression was investigated using nine midlactation cows (193 ± 32 days postpartum; mean ± SD) as previously reported (Harvatine and Bauman 2006). Briefly, cows were arranged in a 3 × 3 Latin square design with 14‐day experimental periods. Specific treatments were control, short‐term administration of *trans*‐10, *cis*‐12 CLA, and feeding a low‐forage, high‐oil diet (LF/HO; 45.9% forage, 3.0% soybean oil, and 1.5% fish oil). The CLA treatment involved a 3 day intravenous infusion of a CLA‐Intralipid emulsion (days 12–14 of experimental period) that supplied 10 g/d of *trans‐*10, *cis‐*12 CLA. The *trans‐10, cis‐12* CLA methyl ester stock (BASF Corporation; Lampertheim, Germany) contained 88.3% total CLA (98% *trans‐*10, *cis*‐12 isomer), 6.8% palmitic acid, 2.7% oleic acid, and 2.0% stearic acid. Methyl esters of CLA were selected as the convenience of the synthesis procedure make them more commonly available in larger quantities and ME of CLA are equally effective as free FA of CLA in reduction of milk fat in the cow (de Veth et al. 2004). Mammary biopsies were performed at the end of treatment, 1–3 h after milking. In all experiments, tissue samples were immediately frozen in liquid nitrogen and stored at −80°C.

### Cell culture

Bovine mammary epithelial cells (MAC‐T) were grown to 80–90% confluence in basal media [DMEM media plus 5 mmol/L sodium acetate, 5 mmol/L l‐glutamine (Glutamax; Invitrogen, Carlsbad, CA), and 20 IU/mL of penicillin and streptomycin (Invitrogen)] supplemented with 10% fetal calf serum (Gemini Bio‐products, West Sacremento, CA) and 5 µg/mL bovine insulin (Sigma, St. Louis, MO). Cells were incubated in basal media in the absence of serum and insulin for 24 h before treatment. Cells were treated for 24 h with 5 *μ*mol/L LXR agonist TO‐901317 (TO9; Calbiochem, San Diego, CA), 10 *μ*mol/L 9cRA (Sigma), or 75 *μ*mol/L t*rans‐*10, *cis‐*12 CLA alone or in combination. Treatments were performed in both basal media and media supplemented with serum and insulin as described in the figure legends. The CLA selected for cell culture was in free FA form to provide the natural form that does not require hydrolysis and contained 95.3% *trans*‐10, *cis*‐12 CLA and 2.2% linoleic acid (Larodan AB, Malmö, Sweden). The CLA stock was complexed to BSA after formation of a potassium salt (Keating et al. 2007). Lipogenesis was determined by ^14^C acetate incorporation into FA as described by Peterson et al. (2004).

### RNA isolation and real‐time PCR

RNA isolation and Real‐Time PCR (qRT‐PCR) was performed according to Harvatine and Bauman (2006) with minor modifications. Briefly, total RNA was isolated using the RNeasy Lipid Kit with on‐column DNase treatment (Qiagen; Valencia, CA), and RNA was reverse transcribed using SuperScript III and random primers (Invitrogen). qRT‐PCR reactions included ABI Power SYBR with ROX (Applied Biosystems Inc, Foster City, CA) and 400 nmol/L of gene‐specific forward and reverse primers (RXR*α* (Mamo et al. 2005), ribosomal protein S9 (Janovick‐Guretzky et al. 2007), and others in Harvatine and Bauman (2006) and Table 1; Invitrogen). Expression level was determined relative to a dilution curve of pooled cDNA as described in Balmer and Blomhoff (2002).

**Table 1.** Primers used in Real‐Time quantitative reverse transcription PCR analysis.

Gene/Primers	Sequence	Accession No.
ABCA1	F: AAGAAGTAGGCAAGGTTGGCG	NM_001024693
	R: GCCCACCAATCAAAGCCAT	
ChREBP	F: GCTGCAGCATGGCAATGTACT	TC350579
	R: GGAGCAGAAGAGGCGTTTCAA	
LXR*α*	F: CATGCCTACGTCTCCATCCA	NM_001014861
	R: TCACCAGTTTCATCAGCATCCT	
LXRß	F: TGCAGCTCGGTCGTGAAG	NM_001014883
	R: CAGCAGCATGATCTCGATGGT	
PRKCSH	F: CTGCACCAACACAGGCTACAA	NM_176662
	R: GTTGTATTCGTCGGTCCCGT	
SREBP1c	F: ATGGATTGCACGTTCGAAGAG	–
	R: CGTAGGGCGGGTCGAATAG	

### Plasmid transfection and luciferase assays

The MAC‐T cells were grown to 85% confluence in 9.6 cm^2^ wells and transfected with 1 *μ*g of luciferase plasmid and 0.067 *μ*g of the control TK renilla plasmid (Promega, Madison, WI) using Lipofectamine 2000 (Invitrogen). Luciferase plasmids included constructs containing approximately a 1.3 kb fragment of the murine SREBP1C promoter or its mutated version lacking the sterol response element (SRE), LXRE, or both (plasmids D, m24, m31, and m34) from Chen et al. (2004). After transfection, cells were maintained in serum and growth factor‐free media for 16 h before treatments were applied as described above. After 24 h in treatment media, cells were harvested by scraping in 400 *μ*L of Passive Lysis Buffer (Promega). Firefly and Renilla luciferase activity was determined using the Dual‐Luciferase reporter assay (Promega) according to manufacturer's protocol.

### Statistical analysis

Data were analyzed using the fit model procedure of JMP (Version 5.0, SAS Institute Inc, Cary, NC) using REML. The model for analysis of tissue expression profile included tissue and the geometric mean of three housekeeping genes [18S ribosomal subunit, ß‐actin, and ß2‐microglobulin (Vandesompele et al. 2002)]. The model for analysis of the lactating and nonlactating mammary tissue included cow (random), lactation state, and the geometric mean of three housekeeping genes (ß‐actin, ß2‐microglobulin, and ribosomal protein S9). The model for analysis of the mammary biopsy experiment included cow (random), period, treatment, and the geometric mean of three housekeeping genes (ß‐actin, ß2‐microglobulin, and ribosomal protein S9). Preplanned contrasts included the effect of CLA (CON vs. CLA) and the effect of the LF/HO diet (CON vs. LF/HO). The model to analyze cell culture experiments included the random effect of replicate experiment and the fixed effect of treatment, and means were separated using a Protected LSD. In all experiments, data were log transformed when residuals were not uniformly distributed and back‐transformed data are reported. Data points with Studentized Residuals greater than 2.5 were considered outliers and excluded from analysis (rarely more than one per variable).

## Results

### Spatial expression of LXR and ChREBP

**Figure 2.**
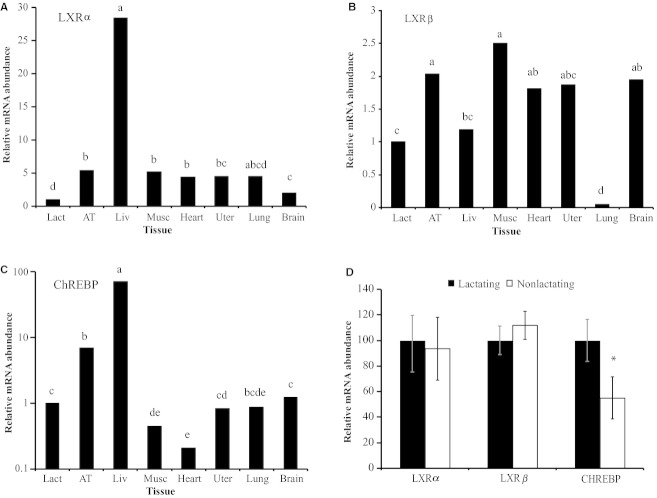
Tissue profile of liver x receptor alpha and beta (LXR*α* and LXR*β*) and carbohydrate response element‐binding protein (ChREBP) in the cow. Panel A‐C: Tissue expression in mid‐to‐late lactation [*n* = 6 for subcutaneous adipose tissue (AT), liver (Liv), and lactating mammary gland (Lact), and *n* = 3 for uterus (Uter), lung, brain, skeletal muscle (Musc), and heart; *n* signifies tissue from different animals]. Panel D: Expression in lactating and nonlactating tissue (*n* = 7 cows sampled in both states). Values represent least‐square means ± SEM. Means are scaled relative to lactating tissue (control set to 100). Panels A, B, and D are linear plots, and Panel C is a semilog plot. Means within panels A to C that differed by *P* < 0.05 are denoted by different letters and difference between lactating and nonlactating tissue in panel D indicated (*P < 0.05).

First, the spatial expression of LXR and ChREBP was investigated to determine if their expression was increased in lipogenic tissues and during lipogenic states. A comparison of tissues from lactating cows indicated that LXR*α* and ChREBP were predominantly expressed in liver, and LXRß was ubiquitously expressed at similar concentrations in all tissues except lung (Fig. 2A–C). Mammary expression of LXR*α* and LXRß was not different between lactating and nonlactating tissue, but ChREBP expression was greater in lactating than nonlactating tissue (*P *<**0.05; Fig. 2D).

### Expression of LXR, RXR, and associated proteins during MFD

**Figure 3.**
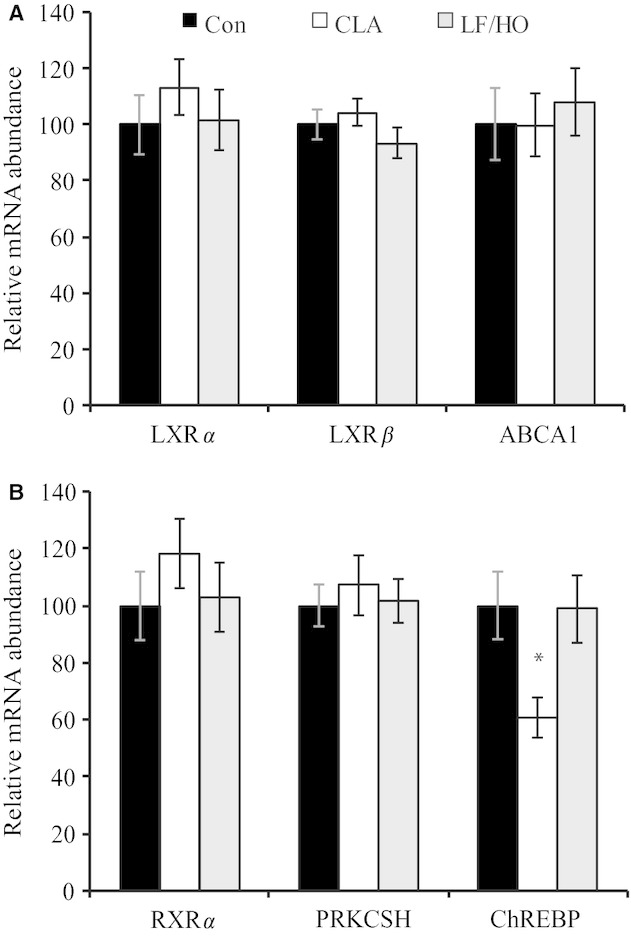
Effects of milk fat depression induced by *trans‐*10, *cis‐*12 conjugated linoleic acid (CLA) or a low‐forage, high‐oil diet (LF/HO) on mammary mRNA abundance in dairy cows. Panel A: Liver x receptor alpha and beta (LXR*α* and LXR*β*) and the LXR‐responsive gene ATP‐binding cassette 1 (ABCA1). Panel B: retinoid X receptor alpha (RXR*α*), the RXR‐responsive gene protein kinase C substrate 80K‐H (PRKCSH), and carbohydrate response element‐binding protein (ChREBP). Infusion of *trans‐*10, *cis‐*12 CLA decreased milk fat concentration and yield by 23 and 24%, respectively, and the LF/HO diet decreased milk fat concentration and yield 31 and 38%, respectively (Harvatine and Bauman 2006). Values represent least‐square means ± SEM. Means are scaled relative to control (control set to 100; *n *=**8–9 samples per treatment). Significant difference between lactating and nonlactating tissue indicated (*P <**0.05).

Next, we determined if LXR, RXR, and ChREBP expression was modified in the mammary gland during nutritional inhibition of milk fat synthesis in the cow. Short‐term infusion of *trans‐*10, *cis‐*12 CLA decreased milk fat concentration and yield by 23 and 24%, respectively, and the LF/HO diet decreased milk fat concentration and yield 31 and 38%, respectively (Harvatine and Bauman 2006). Yields of FA of all chain lengths were decreased during MFD; however, the decrease was greater for de novo synthesized FA resulting in a shift in milk fat composition to an increased proportion of long chain FA (Harvatine and Bauman 2006). Mammary tissue was obtained at the end of the treatment and we have previously reported decreased expression of fatty acid synthase, lipoprotein lipase, S14, SREBP1, and insulin‐induced gene 1 (Harvatine and Bauman 2006), which are regulated by multiple lipogenic factors. In the current analysis, we assayed expression of LXR*α*, LXRß, and the predominantly LXR‐regulated gene ABCA1 in the same mammary tissue samples. Mammary expression of these genes was not affected by CLA‐induced MFD or by diet‐induced MFD (Fig. 3A). Likewise, RXR*α* and the RXR‐regulated gene protein kinase C substrate 80K‐H (PRKCSH) were not altered (Fig. 3B). Lastly, expression of ChREBP was decreased during CLA treatment, but not during MFD induced by feeding a LF/HO diet (*P *<**0.03 and *P *=**0.95, respectively; Fig. 3B).

### Effect of CLA on LXR‐mediated lipogenesis in MAC‐T cells

**Figure 4.**
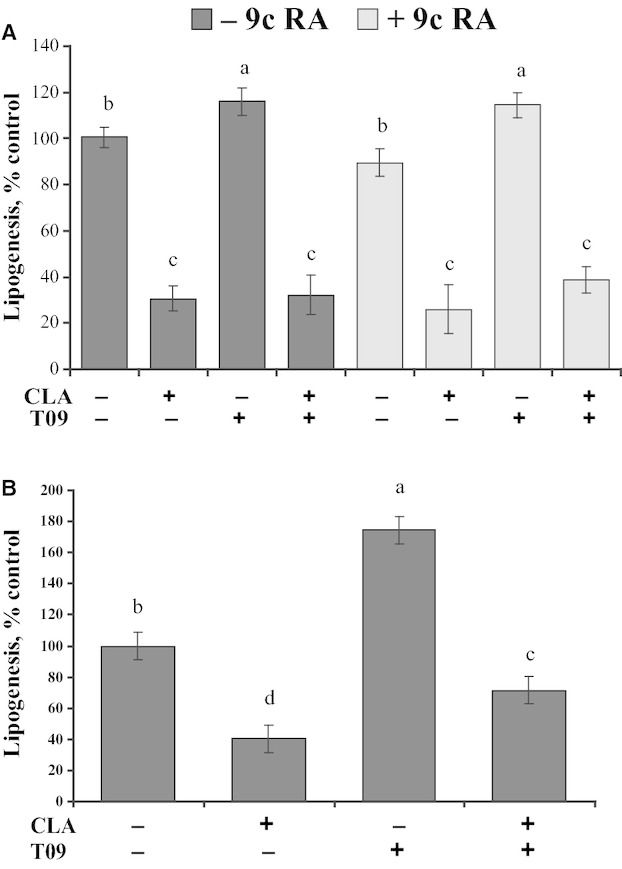
Effect of *trans‐*10, *cis‐*12 conjugated linoleic acid (CLA) and LXR agonist (TO9) on lipogenesis in a bovine mammary epithelial cell line (MAC‐T). Lipogenesis was measured by incorporation of ^14^C acetate into lipids during the last 4 h of treatment and data are expressed as a percent of control. Panel A: Cells were treated for 24 h in basal media with 75 *µ*mol/L CLA, 5 *µ*mol/L LXR agonist (TO‐901317), 10 *µ*mol/L of 9‐cis retinoic acid (9c RA), or combinations of these treatments. Panel B: Cells were treated for 24h in basal media supplemented with 5% fetal bovine serum and 2.5 *µ*g/mL of bovine insulin with 75 *µ*mol/L CLA, 5 *µ*mol/L LXR agonist (TO‐901317), or combinations of these treatments. Values represent least‐square means ± SEM (includes intra‐ and interexperimental run error). Means are scaled relative to control (control set to 100; *n *=**6 wells per treatment across two independent experiments Panel A and *n *=**3 wells per treatment for panel B). Means within panels that differed by *P *<**0.05 are denoted by different letters.

Liver x receptors are regulated by ligand activation requiring further testing beyond changes in gene expression. The ability of an LXR agonist to overcome the effect of CLA was tested in bovine mammary epithelial cell culture. Lipogenesis in MAC‐T cells was increased by LXR agonist (TO9) and decreased by CLA when cultured in basal media or basal media supplemented with serum and insulin (Fig. 4A,B). RXR agonist (9cRA) did not increase lipogenesis in basal media (Fig. 4A). Addition of CLA decreased lipogenesis 69% in basal media and 71, 72, and 66% in basal media plus 9cRA, TO9, and 9cRA + TO9, respectively. CLA decreased lipogenesis in basal media supplemented with serum and insulin by 60% and decreased lipogenesis in basal media supplemented with serum, insulin, and TO9 by 59% (Fig. 4B).

**Figure 5.**
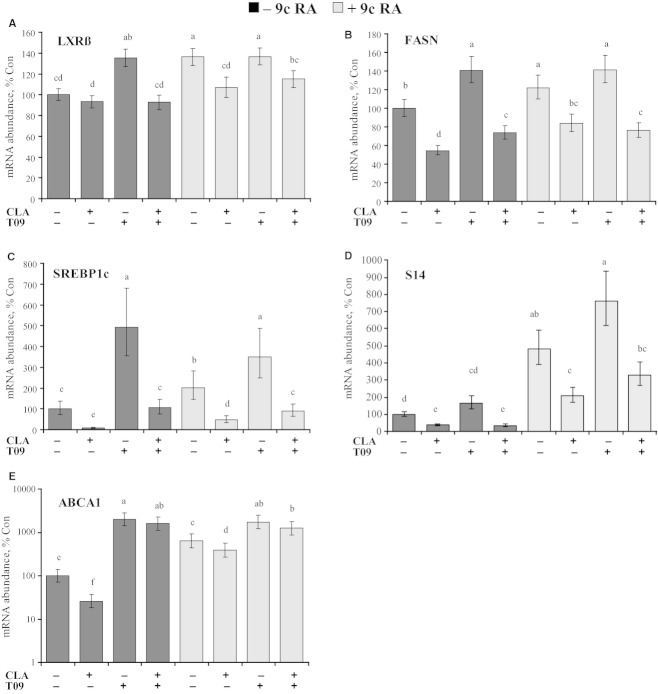
Effect of *trans‐*10, *cis‐*12 conjugated linoleic acid (CLA) and LXR agonist (TO9) on gene expression in a bovine mammary epithelial cell line (MAC‐T). Cells were treated for 24 h in basal media with 75 *µ*mol/L CLA, 5 *µ*mol/L LXR agonist (TO‐901317), 10 *µ*mol/L of 9‐cis retinoic acid (9c RA) or combinations of these treatments. Panel A: Expression of liver x receptor beta (LXRß); Panel B: Expression of fatty acid synthase (FASN) known to be responsive to LXR and SREBP1c in rodent liver; Panel C: Expression of SREBP1c known to be regulated by LXR and SREBP1c; Panel D: Expression of thyroid hormone‐responsive spot 14 (S14) known to be regulated by LXR and SREBP1c; and Panel E: Expression of ABCA1 that is predominantly regulated by LXR. Values represent least‐square means ± SEM (includes intra and inter experimental run error). Means are scaled relative to control (control set to 100; *n *=**5‐8 per treatment). Panel A to D are linear plots and Panel E is a semilog plot. Means within panels that differed by *P *<**0.05 are denoted by different letters.

Expression of direct targets of LXR was observed to determine functional LXR signaling mechanisms in bovine mammary epithelial cells. CLA decreased expression and TO9, 9cRA, and 9cRA+TO9 stimulated expression of FASN, SREBP1c, S14, and ABCA1 (Fig. 5A–E). CLA treatment decreased expression of FASN, SREBP1c, and S14 to a similar extent with or without TO9, 9cRA, or 9cRA + TO9 (e.g., SREBP1c decreased 92% with basal media, 78% with TO9, 76% with 9cRA, and 74% with 9cRA/TO9). However, CLA decreased expression of ABCA1 less in the presence of TO9, 9cRA, or 9cRA/TO9 (74% with basal media, 21% with TO9, 38% with 9cRA, and 28% with 9cRA/TO9).

### Mutation of SRE and LXRE‐binding sites

**Figure 6.**
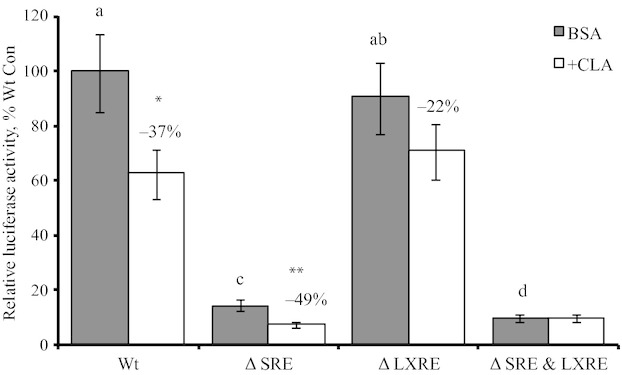
Regulation of the SREBP1c promoter by trans‐10, cis‐12 conjugated linoleic acid (CLA). Luciferase plasmids containing approximately a 1.3 kb fragment of the murine SREBP1C promoter with site‐specific mutations in the sterol response element (Δ SRE), LXR response element (Δ LXRE), or both (Δ SRE&LXRE) from Chen et al. (2004; plasmids D, m24, m31, and m34). Activity of the wild‐type (WT) and mutated promoters was tested in basal media with vehicle control (BSA) or with the 75 *µ*mol/L CLA for 24 h. Values represent least‐sqaure means ± SEM. Means are scaled relative to control (control set to 100; *n *=**6 per treatment). Means that differed during control treatment by *P *<**0.05 are denoted by different letters and inhibition by CLA within each promoter construct is shown along with the percent inhibition relative to control media (*P <**0.05 and **P <**0.01).

The SREBP1c gene is regulated by both SREBP1 and LXR making interpretation of TO9 stimulation of SREBP1 expression more complicated. An SREBP1 reporter assay was used to determine the role of each independently in response to CLA. Mutation of the SRE and SRE & LXRE drastically decreased the activity of the SREBP1 promoter under basal conditions, but mutation of the LXRE alone had no effect (Fig. 6A). CLA deceased activity of the wild‐type and SRE‐mutated promoter, but had effect on the LXRE and SRE & LXRE‐mutated promoters (Fig. 6B). The extent of inhibition of was similar between the wild‐type and SRE‐mutated promoters.

## Discussion

Diet‐induced MFD results in a specific reduction in milk fat synthesis (Bauman et al., 2011). It is a naturally occurring condition in ruminants, but treatment with *trans‐10, cis‐12* CLA induces a similar response in many species including rats, mice, pigs, sheep, goats, and humans (Bauman et al. 2008). Our working model of CLA regulation of fat synthesis in the mammary gland was based on previous observations of decreased mammary lipogenic capacity and decreased mammary expression of lipogenic enzymes and SREBP1 and S14 during MFD in the cow and direct and indirect regulation of lipogenic enzymes by LXR in adipose and liver tissue of rodents (Fig. 1).

The role of LXRs in the mammary gland has not been extensively studied in vivo, although Rudolph et al. 2007 proposed that they may play a key role in the metabolic shift toward increased de novo lipogenesis and expression of SREBP1 during initiation of lactation in mice. In the cow, lipogenesis is negligible in hepatic cells in both lactating and nonlactating cows, whereas mammary epithelial cells have a very high rate of de novo lipogenesis during lactation (Bauman and Davis 1974). However, LXR*α* was predominantly expressed in the liver, whereas mammary tissue expression of LXR*α* and LXRß was low and not different whether tissue was obtained from lactating or nonlactating cows (Fig. 2). Additionally, Mani et al., (2009, 2010) reported a modest increase in LXR*α* expression in early lactation in the cow, but this increase was not sustained throughout lactation. We are not aware of a published investigation of the functional role of LXR on the mammary gland, although LXR null mice would provide a robust test of the functional role of LXRs in mammary development, differentiation, and milk synthesis.

The transcriptional activity of LXRs is regulated at multiple levels including expression, ligand binding, and association with coactivators and repressors. To evaluate regulation of LXR and RXR in the mammary gland of the cow during diet‐induced MFD or MFD induced by treatment with CLA, we first determined expression of the nuclear receptors and select target genes. Identification of target genes is complicated by multiple transcription factors regulating gene expression. For example, SREBP1c and S14 are LXR‐responsive genes, but are also markedly responsive to other factors including abundance of the active nuclear SREBP1c fragment (Fig. 1). ABCA1 is distinctly responsive to LXR and not SREBP1c (Liang et al. 2002; Schmitz and Langmann 2005), although regulation by SREBP1a and SREBP2 has been reported (Tamehiro et al. 2007; Zhou et al. 2008). ATP‐binding cassette G5 and G8 have been classically used as specific LXR‐responsive genes. Viturro et al. 2006 reported expression of both ATP‐binding cassette G5 and G8 in the bovine mammary gland, but we were unable to achieve quantitative expression of either gene in lactating mammary tissue or MAC‐T cells using the primers of Viturro et al. (2006) or primers that we independently designed and validated using bovine liver cDNA. In the present study, mammary expression of LXR and its selected target gene (ABCA1) was not modified by diet‐induced or CLA‐induced MFD. However, LXR agonist increased expression of LXRß and ABCA1 in MAC‐T cells demonstrating functional signaling of LXR in bovine mammary epithelial cells (Fig. 5).

Identification of a gene to assess activation of RXR is challenging as RXR predominantly functions as a heterodimer with numerous nuclear receptors that regulate metabolism including LXRs, PPARs, RARs, FXR, PXR, and COUPTFs. A RXR ligand is required for heterodimer formation, but does not change heterodimer transcriptional activity. However, expression of PRKCSH is dependent on RXR tetramers, which are formed in the absence of RXR ligand (Yasmin et al. 2004). In the current experiment, the lack of change in expression of RXR and PRKCSH in the mammary gland during MFD provides no support for a RXR mechanism.

The ability of CLA to overcome the LXR agonist TO9 and the RXR agonist 9cRA was investigated using a bovine mammary epithelial cell line (MAC‐T). MAC‐T cells were derived from primary mammary epithelial cells from a lactating cow (Huynh et al. 1991); this is the major mammary cell line utilized in research of the bovine mammary gland. In the current experiments, culture media included both acetate and glucose, but the lipogenesis assay was based on acetate utilization. Media glucose concentration was high relative to physiological levels similar to other culture systems, but glucose is essential for synthesis of glycerol and part of the NADPH during lipid synthesis and cannot be eliminated. Based on previous work and the current experiments, CLA reduces expression of lipogenic enzymes, SREBP, and S14 in MAC‐T cells similar to that observed in vivo. Importantly, CLA decreased lipogenesis to a similar extent in the absence and presence of LXR and RXR agonists; therefore, results do not support a LXR‐dependent mechanism for CLA‐induced inhibition of lipogenesis in the mammary gland. The agonists tested have a high affinity for their respective receptors and are expected to out‐compete CLA for ligand binding, thereby blocking any response due to a direct effect of CLA on the receptor. In the current experiment, LXR agonist and CLA were each tested at only one concentration. The dose of CLA was selected based on previous dose titrations and represents near the maximal inhibition of lipogenesis. We also expect that we were above the maximal effective dose of the LXR agonist as others have reported maximal cholesterol efflux at 1 *μ*mol/L of TO9 (Czech et al. 2007). Under basal conditions in the current experiment, LXR agonist increased lipogenesis demonstrating an effective dose of TO9 and functional regulation of lipogenesis by LXR in bovine mammary epithelial cells (Fig. 2) similar to previous reports (McFadden and Corl 2010). Interestingly, McFadden and Corl 2010 reported that TO9 caused cell death at concentrations above 2 *μ*mol/L in a different bovine mammary epithelial cell line (BME‐UV). Cell death was not observed in the current experiment with 5 *μ*mol/L TO9 or in MAC‐T cells treated with 2.5 *μ*mol/L TO9 (Oppi‐Williams et al. 2012) and 10 *μ*mol/L is commonly used in investigation of LXR regulation of hepatocyte lipogenesis without apparent toxicity (Hegarty et al. 2005). The current experiment demonstrates CLA is dominant over TO9 at near the expected maximally effective doses of CLA and TO9. Higher doses of CLA may overcome the LXR agonist, but high doses of CLA are also known to induce other mechanisms including inflammatory pathways (Foote et al. 2010). Importantly, the CLA dose tested in the current experiment decreases lipogenesis and expression of lipogenic enzymes, SREBP1, and S14 similar to that observed in vivo, thus represents the physiological mechanism of interest.

The ability of CLA to reduce mammary expression of key lipogenic regulators, lipogenic enzymes, and LXR target gene in the presence of LXR and RXR agonists was investigated based on our working model (Fig. 1). Signaling experiments were conducted in basal media without serum and insulin supplementation to reduce confounding prolipogenic factors. Both in vivo and in vitro studies have demonstrated that insulin does not stimulate rates of mammary lipogenesis in the lactating cow [see review (Bauman and Griinari 2003)], but the high insulin dose in the current experiment is expected to stimulate the IGF‐I receptor. Increased expression of lipogenic transcription factors and enzymes demonstrates the presence of functional LXR signaling similar to that reported by McFadden and Corl (2010) and Oppi‐Williams et al. (2012). However, Oppi‐Williams et al. (2012) also reported that LXR knockdown did not decrease lipogenesis in MAC‐T mammary epithelial cells. Knockdown of LXR was considered as a second method to investigate the functional interaction of CLA and LXR, but was not conducted in the current experiments do to the lack of data supporting an LXR mechanism in the mammary gland during MFD. In the current experiment, CLA was equally effective in decreasing expression of lipogenic signals and enzymes in the absence and presence of LXR agonist, RXR agonist, and their combination, further supporting an LXR and RXR independent mechanism of CLA in regulation of lipid synthesis. Increased expression of FASN, LXR, SREBP1c, and S14 by 9cRA, but no change in lipogenic capacity in this model suggests posttranslational regulation, although not specifically investigated in the current experiment. S14 was especially responsive to 9cRA supplementation. The exact mechanism of this is not clear, but S14 is a retinoic acid‐responsive gene and RXR*α* was functionally important to S14 expression (Nagaya et al. 1998; Balmer and Blomhoff 2002). Additionally, RXR functions as both a homodimer and heterodimer with numerous nuclear hormone receptors and may demonstrate a deficiency of RXR ligand in basal media.

ATP‐binding cassette 1 is a LXR target gene, but is not regulated by SREBP1c (Liang et al. 2002) and is known to be responsive to 9cRA (Chen et al. 2011). In the current experiment, T09 and 9cRA drastically increased expression of ABCA1 in bovine mammary epithelial cells. Additionally, expression of ABCA1 was decreased by CLA in basal media, but was not decreased by CLA in the presence of LXR agonist. *trans‐*10, *cis‐*12 CLA was not expected to decrease expression of ABCA1, but ABCA1 is regulated by many factors including SREBP1a and SREBP2, and SREBP1a is highly expressed in many cell lines. However, mammary expression of ABCA1 was not decreased in the cow during MFD. Interestingly, *trans*‐9, *trans*‐11 CLA is an LXR agonist (El Roz et al. 2013), but does not reduce milk fat synthesis in dairy cows (Perfield et al. 2007). Rumen fermentation results in synthesis of a wide range of CLA isomers during diet‐induced MFD, but it appears that the CLA isomers which inhibit milk fat synthesis are either not LXR agonists or are not produced in sufficient quantity. Additionally, this may be a cell‐specific response as macrophage activity and expression of ABCA1 is reduced by PUFA (Uehara et al. 2002) including CLA (Wang et al. 2004) through an LXR independent mechanism.

The SREBP1c promoter assay allowed dissection of the SREBP1c response to TO9 and CLA in bovine mammary epithelial cells. The drastic reduction in the activity of the SRE‐mutated promoter demonstrates a predominant role for SREBP1c in its own regulation and the lack of change in activity with mutation of the LXRE questions the importance of LXR in mammary SREBP1 regulation. Treatment with CLA caused the largest reductions in the activity of the wild‐type and SRE‐mutated promoter, although the magnitude of the decrease in the SRE promoter was small due to the low activity of the promoter. However, this does demonstrate the opportunity for a non‐SREBP1c mechanism. Additionally, CLA had no effect on the SRE & LXRE‐mutated promoter demonstrating a possible role of LXR in the CLA response. However, SREBP1 clearly has the predominant role in regulation of the SREBP1 promoter and the potential to explain the magnitude of responses observed during MFD in the cow.

Glucose metabolism and fat synthesis are integrated by activation of ChREBP by xylulose 5‐phosphate, a metabolite of glucose, and activation of ChREBP simultaneously activates glycolysis and lipid synthesis through transcriptional regulation of target genes including l‐pyruvate kinase and FASN (Uyeda and Repa 2006). ChREBP is a LXR target gene and is responsible for a portion of LXR stimulated lipogenesis (Cha and Repa 2007). Additionally, PUFA have been reported to decrease mRNA abundance of ChREBP and its heterodimeric partner MAX‐like factor X (Dentin et al. 2005; Xu et al. 2006). Lastly, Kadegowda et al. (2010) reported that CLA did not effect mammary expression of LXR*α* or RXR*α*, but decreased expression of ChREBP in lactating mice. However, ChREBP protein was not detectable in adipose depleted mouse mammary cells (Rudolph et al. 2009) questioning the functional role of decreased ChREBP in mammary epithelial cells. Mammary tissue obtained from lactating cows has very few adipocytes; we observed that ChREBP was not highly expressed in bovine mammary tissue and is therefore unlikely to be a significant signaling factor for regulating milk fat synthesis. Bovine mammary tissue has a very low rate of glycolysis due to metabolic adaptations to conserve glucose from being used as an energy and carbon source; instead, acetate is the predominant carbon source for lipid synthesis (Bauman and Davis 1974). However, mammary expression of ChREBP was decreased by *trans*‐10, *cis‐*12 CLA treatment, but not during diet‐induced MFD in the current experiment (Fig. 3). Expression of ChREBP was below the level of quantification in MAC‐T cells limiting further investigation of the interaction of CLA and LXR agonist‐stimulated lipogenesis and further questioning the importance of ChREBP in the mammary gland. Although ChREBP is downregulated during CLA‐induced MFD, the lack of a response during diet‐induced MFD and the low expression of ChREBP in mammary tissue suggest that changes in its expression are not required to elicit the observed reduction in milk fat synthesis. However, additional investigations will be required to determine the functional role of ChREBP in bovine mammary tissue and CLA‐induced inhibition of milk fat synthesis.

In conclusion, the low expression of LXRs in lactating mammary tissue from the dairy cow raises questions about the importance of LXRs in milk fat synthesis. The lack of change in expression of specific LXR target genes and the failure of LXR and RXR agonists to overcome *trans*‐10, *cis*‐12 CLA inhibition of lipogenesis does not support major CLA signaling through LXR. Recently, Ducheix et al. (2013) reported that lipid synthesis failed to be upregulated during essential FA efficiency in LXR null mice, demonstrating an in vivo role of LXR in hepatic lipid metabolism. A functional role of LXR in vivo was not tested in the current experiment, but taken together the data do not offer support for an LXR‐dependent mechanism of CLA‐induced milk fat depression in the mammary gland. A functional role of LXR could be further testing using a combination of genetic overexpression and deletion approaches in cell culture or lactating mice. However, based on current evidence future work may be more effectively directed toward other candidate systems responsive to bioactive FA including PPAR's, SREBP1, S14, and ER stress signaling pathways.

## Acknowledgements

The authors would like to specifically recognize the support and technical assistance of D.A. Dwyer. Additionally, the authors acknowledge the assistance of former Cornell students and employees Dr. S. Thorn, Dr. J.W. Perfield, Dr. M. Tanino, Dr. C. Tyburczy, Dr. E. Castañeda‐Gutiérrez, Dr. A.L. Lock, L. Furman, B. English, Dr. W. Waybright, and A. Garcia‐Ramirez.

## Conflict of Interest

None declared.
